# Supplementary material for: Addressing Nutritional Knowledge Gaps in Inflammatory Bowel Disease: A Scoping Review
Source: Nutrients. 2025 Feb 27;17(5):833. doi: 10.3390/nu17050833 (PMC11902248; doi:10.3390/nu17050833)
Supplement: Supplementary file 1 [file nutrients-17-00833-s001.zip › Table S1.pdf]

Table S1. Key characteristics of the included studies

Author and Year	Full title	Country	Type of study	No. Participant	Characteristics of participants	Assessment tools	Nutrition knowledge	Eating behaviors	Results	Conclusion
Shafiee NH et al., 2020	An assessment of dietary intake, food avoidance and food beliefs in patients with ulcerative colitis of different disease status	Malaysia	Cross-sectional	64	UC (64.1% inactive, 35.9% active).	Dietary History Questionnaire, Powell Tuck Index	Low adherence to nutrient recommendations	Avoidance of dairy, high-fat foods; dietary intake inadequate	Active UC patients had poorer intake; both groups below standards	Nutritional education essential to prevent deficiencies
Godala M et al., 2020	Dietary Behaviors and Beliefs in Patients with Inflammatory Bowel Disease	Poland	Cross-sectional	85	IBD patients.	Interviews conducted by a trained interviewer	High rates of food beliefs influencing behavior	Avoidance of raw vegetables, dairy, legumes	81.7% avoided specific foods, many imposed restrictions post-diagnosis	Structured education needed to bridge belief gaps and evidence
Zallot C et al., 2012	Dietary Beliefs and Behavior Among Inflammatory Bowel Disease Patients	France	Cross-sectional survey	244	IBD patients (72.5% CD).	Questionnaire	Limited understanding of evidence-based nutrition	High avoidance of spicy, fatty foods	Beliefs shaped food restrictions, impacting social life	Dietary beliefs impact social and dietary habits; need education
Crooks B et al., 2022	Dietary beliefs and recommendations in inflammatory bowel disease: a national survey of healthcare professionals in the UK	UK	Cross-sectional survey	223	Gastroenterologists (50%) IBD clinical nurse specialists (23%), Dietitians (27%)	Online survey	Inconsistent knowledge among HCPs.	Recommendations to avoid high-fiber, fatty foods	HCPs inconsistently advised dietary changes, need for structured training	Education for HCPs can enhance dietary support consistency
Jowett SL et al. 2003	Dietary beliefs of people with ulcerative colitis and their effect on relapse and nutrient intake.	UK	Cohort study	183	Patients with UC in clinical remission	Food frequency questionnaire, disease activity index	Common belief in dietary impact, but no effect on relapse	High avoidance of milk, dairy products; nutrient intake affected	No significant dietary effect on relapse risk	Education needed to prevent nutritional deficiencies
Uršulin-Trstenjak N et al., 2021	Dietary habits of patients with inflammatory bowel disease from the territory of northern Croatia	Croatia	Cross-sectional study	93	IBD patients (50.5% CD; 49.5% UC).	Questionnaire	Mixed understanding, improved post-diagnosis	Increased meals post-diagnosis; probiotics used	Improved meal frequency; common avoidance of dairy, fats	Dietary habits improved post-diagnosis; education required
Limdi JK, et al., 2016	Dietary Practices and Beliefs in Patients with Inflammatory Bowel Disease	UK	Cross-sectional survey	400	IBD patients, mixed demographics	Questionnaire dietary habits and beliefs questionnaire	Low; many patients self-directed diets	High rates of food avoidance; preference for low-fiber diets	Frequent avoidance of spicy, fatty, raw foods; low dietary support	Clearer dietary guidelines and patient education needed
Walton M, et al., 2014	Do patients living with ulcerative colitis adhere to healthy eating guidelines? A cross-sectional study	UK	Cross-sectional	93	UC patients	24-hour dietary recall, questionnaire.	Limited; reliance on unofficial sources.	Avoidance of dairy, fiber-rich foods during flares.	Energy intake below recommendations; unnecessary exclusions.	Avoidance behaviors limit adherence to dietary guidelines.

Yin, T.-T. et al., 2023	Eating is like experiencing a gamble": A qualitative study exploring the dietary decision-making process in adults with inflammatory bowel disease'	China	Qualitative Study	20	IBD patients (13 UC and 7 CD)	Semistructured interviews	Shaped by traditional beliefs, inconsistent with guidelines	Dietary changes often trial-based or influenced by cultural norms	Decision-making based on trial/error, lacking clear guidelines	Understanding patient decision-making aids tailored interventions
Marsh A et al., 2019	Food avoidance in outpatients with Inflammatory Bowel Disease e Who, what and why	Australia	Prospective cross-sectional study	117	Patients with confirmed diagnosis of IBD (CD n=50; UC n=61; IBD type unspecified n=6).	Structured interviews, nutritional assessments	Low confidence in dietary advice, reliance on internet sources	High avoidance of lactose, spicy foods during active disease	High prevalence of avoidance; lack of trust in dietetic advice	Patients need evidence-based advice to reduce unnecessary restrictions
Lim HS, et al. 2018	Food Elimination Diet and Nutritional Deficiency in Patients with Inflammatory Bowel Disease.	Korea	Survey	104 (food exclusion group: n = 49; food non-exclusion group: n = 55)	IBD patients (CD 58,7%; UC 41,3%)	Structured questionnaire	59% of patients in the food exclusion group held dietary beliefs and modified intake accordingly	59% of patients in the food exclusion group held dietary beliefs and modified intake accordingly	59% of patients in the food exclusion group held dietary beliefs and modified intake accordingly	59% of patients in the food exclusion group held dietary beliefs and modified intake accordingly
Pham T et al., 2021	Immune-mediated inflammatory diseases and nutrition: results from an online survey on patients' practices and perceptions	France	Cross-sectional study	300	Adult patients with RA, AS, PsA, CD, UC or PsO registered in an online patient community	Online questionnaire, statistical analysis	Mixed knowledge; varied diet modifications	Changes driven by personal initiative	Majority changed diets; demand for better nutrition advice	Nutrition education can enhance patient self-management
Fiorindi C et al. 2022	Inadequate food literacy is related to the worst health status and limitations in daily life in subjects with inflammatory bowel disease	Italy	Observational Prospective Study	450	IBD patients from AMICI ONLUS association	Food Literacy Survey (FLS-IT), Newest Vital Sign (NVS)	High prevalence of inadequate food literacy	Limited understanding of healthy dietary choices	Poor food literacy linked to worse health outcomes	Targeted education needed to improve dietary literacy
De Vries JHM et al., 2019	Patient's Dietary Beliefs and Behaviours in Inflammatory Bowel Disease	Netherlands	Cross-sectional survey	294	IBD patients (UC 49.7%; CD 64.3%)	Self-administered questionnaire	Self-taught through experience; limited HCP advice	Avoidance of specific foods like spicy, dairy, carbonated drinks	Patients value diet as disease management tool; gaps in advice	Guided dietary strategies improve outcomes; reduce self-restriction risks

Holt DQ et al. 2017	Patients with inflammatory bowel disease and their treating clinicians have different views regarding diet.	Australia	Cross-sectional	928 patients with IBD; 136 HCPs	IBD patients (CD 2/3; UC 1/3); 46 gastroenterologists, 12 surgeons and 73 dietitians;	Online questionnaire	Patients feel clinicians disregard diet importance	Diverse; avoidance of triggers like fiber, nuts	Disparity in diet beliefs requires consistent, evidence-based communication	Disparity in diet beliefs requires consistent, evidence-based communication
Guida L et al. 2021	Perception of the Role of Food and Dietary Modifications in Patients with Inflammatory Bowel Disease: Impact on Lifestyle	Italy	Cross-sectional	167	IBD patients (48.5% UC; 51.5% CD)	Semi-structured interviews	Based on self-experience rather than counseling	Avoidance of heavily seasoned foods, dairy, fruits	Dietary changes improve symptoms but can cause nutritional deficiencies	Holistic approaches needed to align patient experiences with dietary advice
Czuber-Dochan W et al. 2019	Perceptions and psychosocial impact of food, nutrition, eating and drinking in people with inflammatory bowel disease - a qualitative investigation of food-related quality of life	United Kingdom	Qualitative Study	28	IBD patients (16 CD and 12 UC).	Qualitative interviews, Colaizzi's framework	Limited; lack of guidance at diagnosis	Frequent food avoidance, emotional stress from eating	Diet impacts psychosocial aspects of life, creating barriers to social eating	More focus needed on psychosocial support and evidence-based dietary guidance
Sinclair J et al. 2022	Perceptions, beliefs and behaviors of nutritional and supplementary practices in inflammatory bowel disease	United Kingdom	Cross-sectional survey	80	IBD patients	Questionnaire, chi-square tests	High interest but lack of scientific evidence	Mixed; willingness to try supplements	Disconnect between beliefs and actions in diet; low supplement use	Interventions needed to align beliefs and dietary actions
Nowlin S et al. 2020	Perceptive eating as part of the journey in inflammatory bowel disease: Lessons learned from lived experience.	USA	Qualitative Study	16	IBD patients (12 CD; 4 UC)	Semi-structured interviews, NVivo	Guided by experience, limited professional input	Perceptive eating, trial and error for symptoms	Diet seen as symptom management tool; inconsistent practices	Perceptive eating strategies should be supported with professional guidance
Miglioretto C et al. 2023	What do people with inflammatory bowel disease want to know about diet? The dietary information needs of people with inflammatory bowel disease and perceptions of healthcare providers.	Australia	Qualitative Study	13 HCPs and 29 IBD	IBD patients (18 CD; 11 UC); Dietitian (n=10); Gastroenterologist (n=3)	Thematic analysis, interviews	High demand for dietary information	Diet manipulation for control; online info reliance	Desire for holistic care and better dietary information access	Increased access to dietetic services essential for holistic care
Marsilio, I et al. 2020	A Survey on Nutritional Knowledge in Coeliac Disease Compared to Inflammatory Bowel Diseases Patients and Healthy Subjects	Italy	Cross-sectional study	96 IBD patients, 96 CeD patients, 65 healthy controls	Adults with IBD in remission; CeD group on a gluten-free diet; control group with no gastrointestinal diseases	Moynihan Questionnaire (translated and adapted to Italian)	IBD patients show greater knowledge of nutritional recommendations compared to CeD patients	IBD patients follow healthier diets compared to CeD patients, who tend to focus solely on avoiding gluten	CeD patients have lower awareness of nutritional recommendations compared to healthy controls and are less capable of identifying nutrient sources compared to IBD patients	Nutritional consultation is recommended for CeD patients at diagnosis to improve dietary quality and knowledge. IBD patients consider diet a crucial component in symptom management and follow healthier diets

Palamenghi, L et al., 2024	Food-Related Behavioral Patterns in Patients with Inflammatory Bowel Diseases: The Role of Food Involvement and Health Engagement	Italy	Cross-sectional study	890	IBD patients (50% UC, 50% CD)	Online survey, clustering analysis	Differences in health engagement impact dietary adherence	Food choices influenced by emotional regulation, food involvement	Clusters identified with different health engagement levels and behaviors	Personalized dietary interventions needed based on engagement level
Day, A et al., 2021	Food-related quality of life in adults with inflammatory bowel disease is associated with restrictive eating behaviour, disease activity and surgery: A prospective multicentre observational study	Australia	Prospective multicenter observational study	108	IBD patients (69 UC, 39 CD)	Food-Related Quality of Life, Harvey Bradshaw Index, Simple Clinical Colitis Activity Index, Nine-item Avoidant/Restrictive Food Intake Disorder Screen, Depression Anxiety Stress Scale	Most participants obtained dietary information from the internet (60%) and gastroenterologists (46%)	Restrictive eating behaviors driven by fear of negative consequences and reduced appetite were common	Food-related quality of life was poorer in participants with restrictive eating behaviors and active disease. Previous surgery was associated with higher scores	Proactive interventions to reduce restrictive eating behaviors and improve disease control could enhance food-related quality of life in patients with IBD

Abbreviation: IBD, Inflammatory Bowel Disease; CD, Crohn's Disease; UC, Ulcerative Colitis; HCPs, HealthCare Professionals; RA, Rheumatoid Arthritis; AS, Ankylosing Spondylitis; PsA, Psoriatic Arthritis; PsO, Psoriasis; CeD, Celiac Disease;
